# Suicide Risk in Glioblastoma Multiforme: A Brief Review of Risk Factors, Screening, and Prevention

**DOI:** 10.1007/s11920-026-01711-2

**Published:** 2026-08-12

**Authors:** Nick Waldvogel, Hyo Ryu, Michelle Riba

**Affiliations:** 1 Department of Psychiatry, University of Michigan, Ann Arbor, MI 48109 USA; 2 Department of Radiation Oncology, University of Michigan, Ann Arbor, MI 48109 USA

**Keywords:** Glioblastoma, Cancer, Suicide, Depression, Psycho-oncology, Quality of life

## Abstract

**Purpose of Review:**

Glioblastoma multiforme (GBM) is an aggressive primary brain tumor associated with poor prognosis, rapid functional decline, and psychological distress. An updated review of the literature is lacking, despite that patients with GBM appear to be among the highest risk for suicide among cancer patients. This review summarizes up-to-date evidence of suicide risk factors, screening approaches, and suicide mitigation strategies in patients with GBM.

**Recent Findings:**

Suicide risk is the highest in the first year following GBM diagnosis. It is also associated with older age, male gender, and tumor-related factors such as supratentorial location. Other factors such as neurocognitive decline and poor functional status may also contribute. Evidence emerging from broader oncology populations suggests that demoralization and existential distress may be factors, though these have not been studied in GBM cohorts. Treatment-related psychiatric adverse effects from corticosteroids, anti-epileptic drugs, chemotherapy, and radiotherapy may also influence psychiatric illness and mediate suicide risk. Screening tools such as the Patient Health Questionnaire-9, Hospital Anxiety and Depression Scale, and Colombia-Suicide Severity Rating Scale have been effectively used to screen for suicide in conjunction with clinical interviewing in GBM patients. Evidence-based suicide reduction interventions include psychotherapy, safety planning, lethal means restriction, and caregiver support.

**Summary:**

GBM is a diagnosis that is associated with higher rates of suicide than almost all other cancers, and the extant literature identifies demographic and tumor features that are associated with suicide in this population. Effective interventions exist to identify and support these patients. Future studies should systematically measure risk factors identified in broader oncology populations to identify additional GBM-specific risk factors for suicide.

## Introduction

Glioblastoma multiforme (GBM) is an aggressive, primary brain malignancy associated with poor outcomes and rapid functional decline. The median survival for patients with GBM is approximately 12–15 months despite treatment. [1] The five-year survival rate is below 10%, and the disease course is frequently associated with poor quality of life. Following diagnosis, patients can become demoralized and depressed and may develop suicidal thoughts or die by suicide. Brain and nervous system cancers are among the malignancies associated with the highest suicide risk, and among these cancers, glioblastoma multiforme and other high-grade gliomas have the highest risk of suicide. [2, 3] Among brain cancer suicide deaths, 62.1% occur in those with glioblastoma multiforme. [4]

A comprehensive literature search was conducted across PubMed/MEDLINE and the Cochrane Library with an emphasis on articles published between 2016 and 2026. English-language original research, systematic reviews, meta-analyses, and clinical practice guidelines were included. Case reports and editorials were excluded. The primary search combined terms related to “glioblastoma,” “glioma,” “brain neoplasms,” “intracranial tumor” with terms for suicide and psychological outcomes (“suicide,” “suicidal ideation,” “depression,” “demoralization,” “psychological distress,” “quality of life”). Secondary searches targeted medication-related psychiatric effects (“dexamethasone,” “levetiracetam,” “temozolomide,” “bevacizumab” combined with “neuropsychiatric,” “psychiatric,” “behavioral”), screening instruments (“PHQ-9,” “HADS,” “Columbia Suicide Severity Rating Scale” combined with “brain tumor” or “cancer”), psychotherapeutic and suicide prevention interventions (“cognitive behavioral therapy,” “meaning-centered psychotherapy,” “safety planning,” “suicide prevention” combined with “cancer” or “glioblastoma”), and cancer suicide epidemiology using registry-based data (“SEER,” “cancer registry” combined with “suicide” and “brain” or “cancer”). Certain seminal citations were included when no newer equivalent exists in the last 10 years.

The purpose of this review is to synthesize existing literature to identify patients with GBM at greatest risk of suicide. In addition, we summarize evidence-based evaluation and management strategies to reduce suicide risk and improve quality of life.

## Risk Factors

### Patient Demographics

Several demographic factors have been consistently associated with suicide risk in patients with GBM. Older age has been identified repeatedly across multiple analyses. One analysis of Medicare data found that 82.8% of suicides in those with GBM were by patients 44 years and older, and another study found that intracranial malignant cancers identified older age (60–79 years old) to be an independent risk factor for suicide, with a standardized mortality ratio (SMR; SMR = number of observed outcomes/number of expected outcomes) of 3.54. [4, 5]

In addition to age, gender has also been found to be a risk factor. One surveillance-based study in GBM found that 93.1% of those who died by suicide were male. Other studies have identified male gender as an independent risk factor for suicide in GBM with a SMR of 1.78. These estimates reflect suicide deaths rather than suicide attempts. Many suicide attempts are nonfatal, with mortality varying substantially by method. [6, 7] No studies in the GBM population have accounted for the burden of suicidal ideation and suicide attempts.

Male gender and older age are risk factors for suicide in the general population, and are theorized to be due to use of more lethal methods, particularly firearms. [8] In the general population, women are twice as likely to attempt suicide but men are four times more likely to die by suicide. This is theorized to be mediated by suicide method by demographics: older males are more likely to use firearms, which are highly lethal. Women are more likely to attempt suicide by overdose, which is less frequently successful, a pattern sometimes referred to as the “gender paradox” in suicide, in that women attempt more, but succeed less, and vice versa. These findings may also apply to patients with GBM, but no literature systematically has measured attempts, only completions. [8]

Socioeconomic risk factors have not been studied in the GBM population, though a large SEER analysis of cancer patients found that Medicaid-insured (SMR 1.72), uninsured (SMR 1.66) were associated with significantly higher suicide risk, and another study showed that patients from the lowest-income counties had persistently higher suicide risk even > 10 years post diagnosis. [2, 9]

### Tumor Characteristics

Studies evaluating malignant intracranial tumors- a broader category that includes GBM and other aggressive primary brain neoplasms- have found that supratentorial tumor location is independently associated with suicide. [5] Glioblastoma histology itself has the highest risk among brain tumors (SMR 4.05). [5]

This association may reflect the neurocognitive and functional deficits associated with tumors in this region. Approximately 90% of patients with supratentorial tumors will experience neurocognitive dysfunction. [10] These tumors are associated with higher rates of motor and executive dysfunction and personality change, which is theorized to impact activities of daily living, work, and independence. [4] In a retrospective study, tumors involving the frontal lobe are significantly associated with postoperative depression scores, [11] further supporting that location not only affects neurological functioning, but also psychiatric disease incidence.

### Demoralization

Demoralization is a clinical construct that is not formally recognized as a Diagnostic and Statistical Manual (DSM) diagnosis. It is characterized by subjective helplessness, hopelessness, and a desire to give up, and is distinguished from major depression by the absence of persistent low mood or anhedonia. Although no published studies have specifically evaluated demoralization in GBM, evidence from the broader oncology population suggests clinical relevance. In a study of 430 oncology patients, demoralization occurred in 21% of patients, but demoralization co-occurred with either a mood or anxiety disorder in only 7% of the sample, meaning that roughly two-thirds of demoralized patients did not have an anxiety or mood disorder. In this study, demoralization was associated with a relative risk of 2.0 (CI 1.1–3.5) for suicidal ideation when controlling for mental disorders such as anxiety or mood disorders. [12] Another cross-sectional study of 303 cancer patients in China found that both demoralization and depression were independent risk factors for suicidal ideation (demoralization with odds ratio (OR) of 5.85; depression with OR of 6.68), but these findings were not controlled for confounders. Furthermore, a recent systematic review identified demoralization as a significant risk factor for suicidal ideation in cancer patients. [13] Due to findings such as these, the National Comprehensive Cancer Network (NCCN) Distress Management Guidelines explicitly list existential distress/demoralization as a distinct entity and recommends screening for and addressing this concern. [14]

### Timing and Prognosis

Suicide risk does not appear to follow a linear trajectory after GBM diagnosis. Multiple studies have identified the highest risk to be within the first year of diagnosis, with risk generally decreasing with time. One study identified a SMR of 3.05 (95% CI, 2.04–4.37) within the first year, compared with 1.17 (95% CI: 0.82–1.63) thereafter. [4] Similarly, another study found that death by suicide was highest in the first year after diagnosis (SMR = 13.04) although this was not an independent predictor after adjustment. [5] In contrast with these findings, a prospective cohort study in brain cancer patients found suicidal ideation peaked between 6 and 9 months post-diagnosis, suggesting that suicidal ideation may actually increase several months after diagnosis. [15]

In the GBM population, several explanations have been proposed for the apparent decline in risk over time. These include the psychological shock from initial diagnosis, improved psychosocial support with time, improving symptom management, and methodological concerns such as competing mortality risk. [16, 17 In dementia patients, progression of disease with physical and cognitive decline has been theorized to be protective against suicide, though this has not been considered in the GBM population. [18] By extension, cognitive decline and delirium, which occur in later stages of GBM, may also contribute to decreased suicide risk in this population.

### Mental Health Diagnoses

In the general population, experiencing a mental disorder increases the risk of suicide. [19] Accordingly, comorbid psychiatric illness may further increase suicide risk among patients with GBM. In one prospective observational study of patients that excluded those with prior psychiatric diagnoses, suicidal ideation was significantly associated with higher depression scores, but suicidal ideation also commonly occurred in those without clinical depression. [15] Clinicians should recognize that suicidal ideation may occur independently of major depression and that screening specifically for suicidal ideation is necessary.

### Medication-Related Psychiatric Effects

GBM patients are frequently treated with medications associated with psychiatric sequelae. For instance, dexamethasone is commonly used to manage peritumoral edema. Dexamethasone may produce dose-dependent insomnia, irritability, depressive symptoms, and psychosis. The ABCD trial studied oncology patients receiving dexamethasone 8 mg twice daily for 1 week, then 4 mg twice daily for 1 week. These patients experienced an increase in neuropsychiatric adverse effects in 18% of cases versus placebo. [20] In addition, GBM patients are highly susceptible to seizures, and anti-epileptic drugs (AEDs) are commonly used. While many of the AEDs are associated with psychiatric side effects, levetiracetam is among the most clinically relevant. It frequently produces non-psychotic behavioral symptoms including irritability, agitation, depression, emotional lability, with a relative risk increase of 7% versus placebo per FDA labeling. [21]

Temozolomide (TMZ) is the standard chemotherapeutic agent for GBM. In animal models, TMZ and radiotherapy have produced anxiety- and depressive-like behaviors in mice, but in humans retrospective studies in high-grade gliomas suggested that concerns regarding TMZ’s cognitive effects are more likely due to tumor progression than TMZ itself. [22, 23 Bevacizumab is FDA approved for recurrent GBM, but it has been associated with deterioration in mood and cognition vs. placebo. [24] In GBM patients, radiotherapy is associated with neuropsychiatric symptoms, though these results are highly confounded by the fact that larger and higher-grade tumors are more likely to receive radiotherapy, and are independently associated with neuropsychiatric effects.

### Evaluation and Treatment

#### Screening and Evaluation

In GBM patients, the Patient Health Questionnaire-9 (PHQ-9) and Hospital Anxiety and Depression Scale (HADS) are the most extensively studied and validated tools for screening for anxiety and depression in this population. [25] Notably, adjusting the positive cutoff from the generally accepted 8 + scoring to 10 + optimized sensitivity and specificity.^25^ In the broader cancer population, increasing the cutoff has been shown to be more accurate as well. This is to mitigate the false positives driven by cancer-driven somatic symptoms such as fatigue, sleep disturbances, appetite changes, and concentration difficulties that GBM patients experience from the cancer rather than specifically due to depression. [25]

As noted above, suicidal ideation occurs in this population in the absence of depression, so specific suicide screeners should be utilized, such as the Columbia-Suicide Severity Rating Scale. [15]

General best-practice recommendations include screening at regular intervals, increasing screening frequency during high-risk periods or with high-risk patients. No screener is perfect and may produce false negatives may occur. Screeners should always be paired with direct clinical questioning, and if feasible, discussion with collateral family members, and by multiple team members.

### Suicide-Reduction Strategies

As aforementioned, older men with GBM appear to be at higher risk; this is also the population that is most likely to own firearms. Lethal means counseling is strongly recommended as it improves firearm locked-storage practices. Firearms account for approximately 50% of suicides in the United States and have a case fatality rate of 85–90% [26]. Even if a patient attempts suicide by another method, other means are far less lethal. Suicide safety planning is also one of the most evidence-based brief interventions for suicide prevention. This includes identifying warning signs, internal and external coping strategies, social supports, crisis contacts, and lethal means counseling; a systematic review found that brief interventions incorporating safety planning were the most consistently associated with reduced suicide attempt rates among interventions.[27]

### Treatment

For those patients who are screened, interviewed, and diagnosed with major depressive disorder, treatment may include psychotherapy and pharmacologic management. No randomized controlled trial has evaluated pharmacologic management of depression in patients with primary brain tumors, a notable gap in the literature. In fact, a 2023 pooled analysis of greater than 1700 patients found antidepressant use at chemotherapy maintenance cycle 4 was associated with poorer outcomes. [28] Another large review found that antidepressants were associated with worse survival after adjusting for covariates. [29] Both studies suggest that antidepressant use is a marker of disease severity rather than a cause of poor outcomes, such as disease progression and worsening functioning. [28, 29]

Depression itself has been shown to be independently associated with overall survival in GBM patients; patients prescribed antidepressants are those with worse survival, making it difficult to distinguish medication effects from confounding by indication. There is no outstanding high-quality evidence evaluating the effectiveness of antidepressants in improving quality of life of GBM or brain tumor patients.

Psychotherapy modalities have been shown to be effective in depressed patients with GBM and cancer in general. In GBM patients, cognitive-behavioral therapy (CBT) delivered by nurses significantly reduced anxiety and depression compared to routine care. [30] In a more generalized cancer population, a RCT of 253 patients reported that Meaning-Centered Group Psychotherapy (MCGP) significantly improved spiritual well-being, quality of life, and decreased depression, hopelessness, and desire for hastened death as compared supportive group psychotherapy. [31]

Applying these findings, a 2024 RCT directly compared MCGP to individual CBT in cancer patients and found MCGP was more effective in improving meaning in life, purpose, and life goals, and equally effective as CBT in improving depression in cancer survivors. [32] Taken together, some data directly exhibits evidence for CBT in GBM, and other modalities, such as MCGP, have been shown to be effective in patients with cancer generally.

## Discussion

Glioblastoma multiforme is a life-changing diagnosis that significantly increases the risk of suicide, to a greater extent than many other cancers. Some features that are broadly associated with suicide in the broader population, such as demographic factors of male gender, older age, and depression are not unexpected. In the GBM population, tumor-specific features such as supratentorial location, and symptom-related features such as demoralization are notable. A particularly high-risk period is within the first year of diagnosis; some data suggest that the risk increases over this first year of diagnosis.

Screening with validated scales for both depression and suicide is imperative, noting that suicidal ideation is known to be a risk even in the absence of depressive symptoms. Validated scales may require higher positive cutoffs due to the somatic symptom overlap of brain tumors with depression. A multidisciplinary treatment approach with psychotherapy, medication management, adequate activity, and supporting caregivers should be considered.

There are significant gaps in the literature regarding suicide risk in patients with GBM. Most studies are retrospective reviews and occasionally lack clinically significant variables. For example, demoralization appears to be a risk factor for suicide in cancer patients, but these factors have not been measured in those with GBM specifically. Additionally, more robust prospective studies are warranted particularly to assess the benefit of antidepressants. While theoretically antidepressants should produce similar outcomes of other populations, a high-quality investigation has not confirmed this.

A recent publication landscape analysis of the last 25 years of GBM research noted that researchers are relatively less interested in the psychological effects of GBM relative to molecular and treatment research. [33] Although continued advances in treatment remain essential, attending to the psychological status of these patients and their families in the present remains equally important. Future prospective studies ought to evaluate psychological variables systematically (including demoralization) to determine their impact on depression, quality of life, and suicide risk in those with GBM.

A recurrent theme in the GBM literature is that poor functioning and prognosis are associated with suicide risk. In the broader oncology setting, demoralization is associated with suicidal ideation. Patients are afraid of becoming worse burdens with time, and many ultimately require increasing levels of support. This may be why demoralization, in the absence of mood or anxiety disorders, has been shown to be a risk factor in other cancer populations. [34] Depression is a disease of distorted, negative thinking, but in some, suicidal thinking may be influenced less by depressive cognition and more by perceived loss of autonomy, progressive disability, with resultant existential distress.

## Conclusion

GBM is a diagnosis that is associated with higher rates of suicide than almost all other cancers, and the extant literature suggests there are demographic and tumor features that are associated with suicide in this population. Effective interventions exist to identify and support these patients. Future studies should systematically measure risk factors identified in broader oncology populations to identify additional GBM-specific risk factors for suicide.

## Key References

Saad 2020, Zhou 2022, Hu 2023

- Saad AM, Elmatboly AM, Gad MM, et al. Association of Brain Cancer With Risk of Suicide JAMA Netw Open. 2020;3(5):e203862. doi:10.1001/jamanetworkopen.2020.3862.
  - ◦This study is the only large SEER study focused exclusively on brain cancer and suicide, linking GBM-specific factors to suicide risk.
- Zhou Z, Jiang P, Zhang P, et al. Incidence, trend and risk factors associated with suicide among patients with malignant intracranial tumors: a surveillance, epidemiology, and end results analysis. *Int J Clin Oncol*. 2022;27(9):1386-1393. doi:10.1007/s10147-022–02206-9.
  - ◦This study has a longer time horizon and broader population of patients, with findings that support theories found in Saad et al.
- Hu X, Ma J, Jemal A, et al. Suicide Risk Among Individuals Diagnosed With Cancer in the US, 2000-2016. *JAMA Netw Open*. 2023;6(1):e2251863. doi:10.1001/jamanetworkopen.2022.51863.
  - ◦Largest and most comprehensive US study of cancer-related suicide, which compares brain cancer malignancy suicide risk to broader cancers.

## Data Availability

No datasets were generated or analysed during the current study.
